# Mathematical processing of RGB data in microfluidic paper-based analytical devices

**DOI:** 10.1038/s41598-024-63546-2

**Published:** 2024-06-13

**Authors:** Marta Fiedoruk-Pogrebniak

**Affiliations:** Faculty of Chemistry, University of Warsaw, Pasteura 1, 02-093 Warsaw, Poland

**Keywords:** Bioanalytical chemistry, Imaging studies, Microfluidics

## Abstract

Microfluidic paper-based analytical devices often are combined with scanners as detectors. In this work, different scanning options offered by scanners: resolution, scanning mode, exposure to radiation, colour restoration, and saving format were tested. Moreover, different attempts to mathematical data treatment based on intensities of three channels—Red, Green and Blue, were studied. All measurements presented in this article were conducted for a model dye—bromothymol blue and a model analyte—zinc(II) ion (complexed with xylenol orange in a paper matrix). The article summarizes the scanning options and possibilities of mathematical calculations. Nevertheless, it is suggested that the best option is to use the prior prepared calculation file to paste obtained intensities and compare all presented in this article (and the most frequently used) equations to process intensities and decide which one should be used in the particular analysis.

## Introduction

Nowadays, microfluidic paper-based analytical devices (µPADs) are gaining more and more interest as they are miniature, simple, portable, fast and economic. µPADs offer a significant reduction of (1) instrumentation costs (2) reagent costs (3) analysis time, (4) volume of consumed samples. Moreover, they are available almost all over the world and are one of the most rapidly developed tools in the field of analytical chemistry, recently. An increasing number of scientific articles about µPADs confirms scientific interest in this topic^1,2^. Paper-based systems enable conducting measurements in the outside laboratory mode as *point-of-care* and *in the field,* what is precious cause the collection, storage and transport may change the compounds forms^3,4^. Moreover, the results are mostly available promptly so it saves also the time which is a priceless value nowadays.

Requirement of using the developed systems outside lab forces proposing new portable solutions for detection—compact, mobile detectors. As the optical detection is the most popular one for µPADs, the commonly used smartphones’ cameras^5^, CCD cameras^6^, detectors made of light emitting diodes and/or photodiodes^7,8^, and scanners are used^9^. Scanning as detection method for microfluidic paper-based analytical devices provides many possibilities to conduct detection and interpret the results. The market offers variety of scanners, among which there are also the portable ones which do not need to be plugged into an AC outlet to work. Most of them are powered via a USB tether connected to laptops, while a few of them have batteries which allows (after fully charging) for scanning up to 1000 pages. Combining the battery-charged ones with the possibility of using memory cards instead of sending received images through Wi-Fi (e-mail, to the cloud) or USB cable to the computer immediately after scanning, makes scanners even more attractive for outside lab measurements. In that case, the computer might be sometimes unnecessary at the measuring site, or can be replaced by smartphones equipped with a dedicated software and/or apps. Moreover, due to the very fast technical development and global trend which miniaturization is, providing portability also among “office” instruments while maintaining very good parameters is crucial. That is why the modern scanners offer high resolution of images (see Table S1 in the Supplementary material). This is important in view of receiving reliable analytical information. However, it is important to remember that higher resolution of received images—longer time of scanning, so the optimum should be chosen between analysis time and resolution of the images needed. Furthermore, as it was mentioned elsewhere^10^ the higher resolution of scanning is not equal to the higher sensitivity. Some models of scanners offer scanning directly into applications such as Adobe Photoshop or Microsoft PowerPoint. Furthermore, some of them allow for scanning small, thick objects as ID cards or laminated cards thanks to dedicated slots in the instruments. This feature is very important for analytical chemistry where the scanning of small pieces of laminated µPADs is performed.

Obtained images (scans) are usually analysed in programs such as Adobe Illustrator, Adobe Photoshop, GenePix, DigitalColor Meter, Corel PhotoPaint and ImageJ^9,11^, whereas the last one is more commonly used (see also Table 1). There are several colour models e.g. RGB, CMYK, HSI, HSV used for digital image processing. Among them the most favoured is the RGB model, which is an additive model based on the perceptive properties of the human eye. All visible colours in this model are created by the additive synthesis and are the result of mixing the intensities of three primary colours—Red, Green, and Blue. Two extreme colours (black and white) in RGB colour space are described as (0, 0, 0) for black and (255, 255, 255) for white as R, G, B, respectively. Using this colour model does not require any additional, mathematical work on the received data because the software “gives” exactly the mean values of components (red, green and blue, at least)^10,12^.

**Table 1:** The review of methods used for colour analysis in RGB model.

Analyte	Colour-forming reagent/reaction	Detector	Analytical signal	Software	References
Ammonium	Nessler's reagent	Smartphone	ED	Dedicated app for smartphone	^13^
${\text{NO}}_{2}^{ - }$, ${\text{NO}}_{3}^{ - }$	Griess reagent, zinc suspension	Scanner	G	ImageJ	^14^
As(III), As(V)	Au(III) chloride, sodium borohydride	Scanner, 1200 dpi	G ($- log\frac{I}{{I_{0} }}$)	ImageJ	^15^
Cu(II)	1-(2-pyridylazo)-2-naphthol	Scanner	ED	Photoshop	^9^
Hg(II)	Colour reaction with AgNPs	Digital camera	RGB intensity	Image processing software	^16^
Formaldehyde	Hantzsch reaction	CMOS camera	B	Self-written RGB analysis software	^17^
Benzoic acid	Janovsky reaction	CMOS camera	R + B	Self-written RGB analysis software	^18^
Cysteine, homocysteine	1,5-diphenylcarbazide-capped AgNPs	Digital camera and mobile camera	Mean gray	ImageJ	^19^
Isoniazid	Methyl orange	Scanner, jpeg, 600 dpi	B	In-house algorithm (MATLAB)	^20^
Alkaloid drugs	Zn-TPP, methyl orange, bromocresol green, Chen’s, iodoplatinate, Dragendorff’s	Scanner, 300 dpi	ED	ImageJ	^21^
Glucose, lactate	GOx, LOx, HRP, 4-AAP, DHBS	Scanner, jpeg, 600 dpi	ED	ImageJ	^22^
Creatinine	Jaffé method	Smartphone, OpenCamera application, jpeg	Hue	ImageJ	^23^
	DNABA method		G		
Albumin	Biuret, Lowry, bicinchoninic acid, Bradford, bromocresol green, tetrabromophenol blue	Smartphone camera	Single colour (R or G)	ImageJ	^24^
Albumin	2'-hydroxychalcone derivatives	Smartphone camera	G/R value	ImageJ and Palette Cam	^25^
Alkaline phosphatase	Pyrophosphate (ALP), thiocholine (BChE), Cu2^+^, o-phenylenediamine, carbon dots	Smartphone camera	G/B value	Color Recognition Application	^26^
Butyrylcholinesterase			$\frac{R + G + B}{R}$		

*ED* Euclidean distance, *R* red, *G* green, *B* blue.

Griess reagent—sulfanilic acid, α-naphthylamine/acetic acid; Zn-TPP—5,10,15,20-Tetraphenyl-21H,23H-porphine zinc; Chen’s reagent—acetic acid, copper(II) sulfate/NaOH; Dragendorff’s reagent—potassium iodide, bismuth subnitrate/acid; GOx—glucose oxidase; LOx—lactate oxidase; HRP—horseradish peroxidase; 4-AAP—4-aminoantipyrine; DHBS—sodium 3,5-dichloro-2-hydroxybenzenesulfonate; DNABA—3,5-dinitrobenzoic acid.

Table 1 presents different data analysis methods based on colour intensities used in µPAD format. In most articles there was no information why the applied method was selected. In some of them the short information, like “based on experimental results” or “the highest intensities”, or “complementary colour” was given. Nevertheless, it does not suggest which and how many methods were considered and examined.

The another colour space often used for the colours description in digital images is the HSV. It is based on three components: Hue (colour; measured in degrees from 0 to 360), Saturation (colour depth) and Value (brightness), both described in the range 0–100%. This model seems to be more adequate in colour characterization because it specifies the colour in an intuitive manner. In the HSV model there is a separation between *chroma* (pure colour) from *luma* (lightness). Nevertheless, the HSV model is more time- and labour-consuming (as it is needed to do recalculations from RGB to HSV values)^23,27^. There is also a third attempt to image analysis—the grayscale. Nevertheless, it is rather rarely used as only luminance is considered^28^.

On the other hand, the RGB colour space has been used in conventional photography and is recommended to save files for presentation on screens. Using the unchanged RGB model (without manipulation of the raw data before the actual analysis) shortens the analysis time and facilitates data treatment. Moreover, it favours the creation of applications that convert RGB data into analytically useful information automatically. As it was presented elsewhere^29^, the comparison of calibration curves, and at the same time sensitivities, intercepts and R^2^, proves that for digital image processing RGB colour space (analysed only as single colours) may give better results than HSV model and grey intensities. Furthermore, most of the scanners support RGB colour, among other formats, what strongly promotes using the RGB colour space in image analysis in analytical chemistry while working with µPADs.

It is worth mentioning that Hamedpour et al.^20^ has proposed a very interesting way of an automatic image processing. In that method several factors, e.g. diameter of inlet and indicator areas, areas of sensing zone and channel, and volumes of sample, methyl orange and buffer, and the interactions between them have been studied. Using machine learning it was possible to calculate the optimal design of the analytical paper-based device allowing receiving the highest predicted colorimetric response. However, all the calculations were done using only the blue colour intensity (which gave the highest value obtained).

An article worth attention on the topic of using scanners as detectors explains in details how office and portable scanners work as well as where the intensities of the most common values (R, G and B) comes from and what is a resolution^10^. Moreover, it focuses on a wide analysis of different parameters offered by scanners, their possibilities as well as the influence of settings on the received sensitivities and coefficients of determination. Furthermore, in the article the influence of the lamination (its occurrence and type of pouches used) or template colour have been studied. Uhlikova et al. has listed parameters and aspects of conducting scanning which does not affect the dynamics of the calibration curve and pointed out those which influence is crucial. This article gives practical guides for performing scanning.

In this article the comparison of different scanning methods using an office scanner for the colorimetric detection is presented. Moreover, the impact of data processing manner and picked method for mathematical analysis on the final results are shown. Bromothymol blue as a model dye and zinc(II) ion as a model analyte has been chosen to present and compare results obtained using different methods of data analysis. All the mathematical calculations have been done in a dedicated calculation sheet.

## Methods

### Reagents

All reagents used were of the analytical grade. Xylenol orange disodium salt (XO, product no. 52097) and zinc chloride (product no. 208086) were purchased from Sigma-Aldrich (USA). Sodium tetraborate, bromothymol blue (BTB), acetic acid, sodium acetate, were purchased from POCh (Poland). For all experiments doubly distilled water was used. Stock solutions of zinc(II) ions were prepared in distilled water, whereas the stock solution of BTB was prepared in 0.01 mol/dm^3^ borate buffer, pH = 9.2. XO solution (5 mmol/dm^3^) was prepared in 0.01 mol/dm^3^ acetate buffer (pH = 4.4). Standard solutions of bromothymol blue and zinc(II) ions were diluted from the appropriate stock solutions.

### Paper-based analytical devices

**Figure 1:**
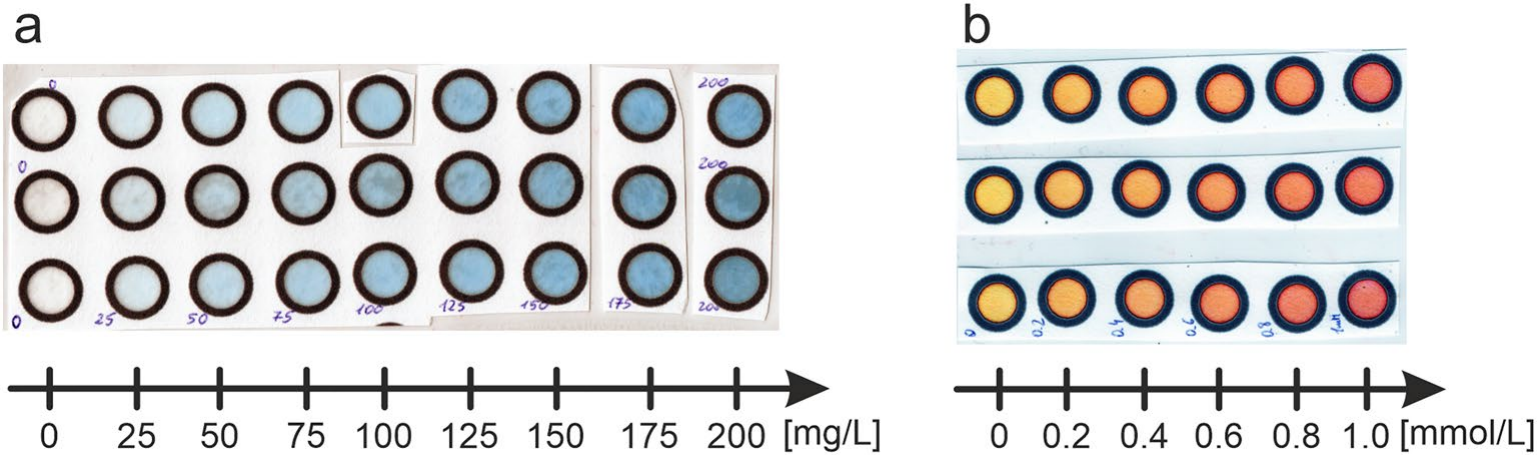
Scans of µPADs with bromothymol blue in borate buffer, pH = 9.2 (**a**) and zinc(II) ions after the reaction (in paper matrix) with xylenol orange, pH = 4.4 (**b**). Concentrations given below arrows refer to BTB and zinc(II) ions.

All measurements were conducted using only detection zones in the shape of circles of 10 mm diameter and 1 mm of borders width. The qualitative filter paper—Whatman 1 (product no. WHA1001185, Sigma-Aldrich, USA) was used throughout. Detection zones were drawn in the CorelDraw software and printed using the solid-ink printer (Xerox ColorQube 8580), and black wax ink (Cartridge-Free ColorQube Ink, Xerox, product no. 108R00966). The printouts were heated in a standard laboratory oven (Alpina easyline EG 40) in the temperature of 115 °C for 60 s as described elsewhere^8^. This procedure ensures obtaining tight hydrophobic barriers. For each concentration three circles were used and 8 µL of solutions were deposited on every filter paper (see Fig. 1). This volume was selected as the optimal one for the proposed geometry of the detection zone in the previous research^8^. In case of BTB solutions just 8 µL of standards were pipetted on the filter paper. Whereas, for zinc(II) ions detection, firstly, 8 µL of the solution of xylenol orange was deposited on the µPADs and left for air drying. Following, 8 µL of the standard solutions of zinc(II) ions were pipetted on detection zones. After 15 min of air drying, scanning was performed. It is worth to mention that i.e. a time gap between standard/sample deposition and detection is also one of the crucial optimization parameters while working with µPADs (this time gap was chosen experimentally, the referenced data are presented in Figure S1.

### Detection

Detection was performed using an office scanner (Epson L3151). During the studies different parameters allowed by the scanner were tested. Filter paper pieces were glued on the cold laminating pouches to avoid moving papers during analysis. The top covers of laminating pouches were not used. Detection was performed after ca 2 min or 15 min following standards deposition for BTB and zinc ions, respectively. The waiting time was chosen experimentally.

For signal reading the ImageJ software (National Institutes of Health, USA) was applied and a “region of interest” (ROI) has been chosen as the whole circle inside hydrophobic barriers throughout. The mean values of R, G, and B channels have been utilized for all data treatment.

## Results and discussion

### Scanning parameters

The office scanner used as a detector offers different work parameters. Among them, the studied were: (1) resolution – 300/600 dpi (as the most popular ones), (2) scanning mode—photo/document, (3) exposure to radiation—low/medium/high, (4) colour restoration—yes/no, (5) saving format—jpeg/tiff. Scans to assess scanning parameters and their impact on the obtained results, including sensitivity, were performed for BTB standard solutions. The images obtained with different scanning parameters are presented in Supplementary material (Fig. S2). Signal analysis was conducted using the “red” values as it is a complementary colour (the closest to orange) to BTB blue looking at the colour wheel (see Fig. S3). Obtained calibration curves are presented in Supplementary material (Fig. S4). Whereas sensitivities are extracted and given in Table 2.

**Table 2:**
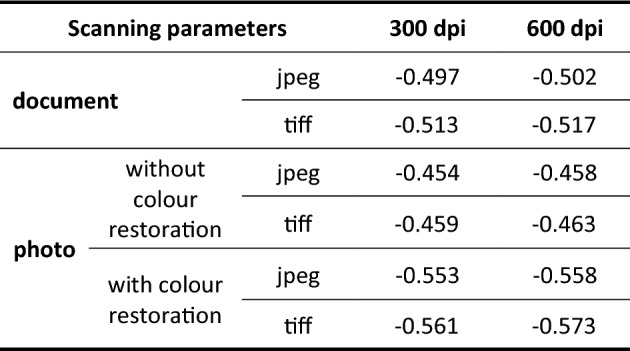
Sensitivities comparison of calibration curves obtained using different scanning methods for BTB standard solutions; slopes given in [L/mg].

It was expected that scanning in the document mode would give the lowest values of sensitivities (see Table 2). Surprisingly, the obtained slopes are much lower for scanning performed in the jpeg format without colour restoration for both resolutions studied. Looking only at sensitivity received for the photo scanning mode it is easy to deduce that scanning performed with colour restoration option gives more than 20% higher value of slope for both jpeg and tiff saving formats, and for both tested resolutions. It indicates the importance of this parameter while performing detection using scanners. Moreover, comparison of the sensitivities received for document and photo modes (with colour restoration option) of scanning clearly indicates that photo gives higher sensitivities. Interestingly, scanning in 300 dpi and 600 dpi, and jpeg-tiff as saving formats influences the obtained sensitivities not as much as it was expected. Nevertheless, for further µPADs signal detection with using scanners, as the most promising parameters, scanning in the photo mode, with colour restoration, tiff saving format were selected. However, if the size of the images is crucial, then saving in jpeg format is recommended what stays in agreement with only recently published practical guide^10^.

### Mathematical data treatment

In scientific literature (see Table 1), many examples of performing data analysis after colour detection, especially in RBG colour space are presented. This way of analysis is first of all used for detection implemented using scanners, cameras and smartphones’ cameras. Equations which are frequently employed for calculations of analytical signal (AS) in RGB model are listed below—equations from (1) to (7).

1 $$AS = \frac{R + G + B}{3}$$

2 $$\begin{aligned} AS = & - log\frac{I}{{I_{0} }}\quad \left( {{\text{for R}},{\text{ G or B}}} \right) \\ & I {-}{\text{ intensity }}\left( {{\text{of R}},{\text{ G or B}},{\text{ respectively}}} \right) \\ \end{aligned}$$

3 $$AS = \Delta I = I_{0} - I_{reaction} \quad \left( {{\text{for R}},{\text{ G or B}}} \right)$$

4 $$AS = \Delta R + \Delta G + \Delta B$$

5 $$AS = \frac{R + G + B}{R}$$

5' $$AS = \frac{R + G + B}{G}$$

5'' $$AS = \frac{R + G + B}{B}$$

5''' $${\text{or e}}.{\text{g}}.\quad \quad AS = \frac{G}{B}$$

6 $$\begin{aligned} AS = ED = & \sqrt {(\Delta R_{1} )^{2} + (\Delta G_{1} )^{2} + (\Delta B_{1} )^{2} + (\Delta R_{2} )^{2} + \cdots } \\ & \quad named\; \, also\; \, as\;{ ``}RGB \, \;distance^{\prime\prime} \\ \end{aligned}$$

7 $$\begin{aligned} AS = & - \gamma log\frac{RGB}{{R_{0} G_{0} B_{0} }} \\ & \gamma - {\text{ correction factor }}\left( {{\text{may be used}},{\text{ not necessary}}} \right) \\ \end{aligned}$$

In order to select one optimal colour variant for data analysis and preparation of calibration curve, one of two options is most often implemented. The first one assumes the preparation of separate calibration curves for each colour variant (usually done only for single colours from the RBG model) because of the time-consuming procedure. On the other hand, the most popular, the easiest and much faster way in data preparation is to use only one colour (its intensity) to create calibration curve. This option assumes selection of the complementary colour at the colour wheel to the one obtained after colour reaction and take only intensity of that colour to create calibration graph.

Nevertheless, among published articles (see Table 1) it is found that only one method is used for particular analysis. Moreover, in most of publications there is neither explanation, nor proof why “this”, not “that” method has been chosen. In this article several calculation methods have been applied for making calibration curves for bromothymol blue and zinc(II) ions (after reaction with xylenol orange) to be able to prepare proper comparison of these methods. In Fig. 1 scans of the standard solutions of bromothymol blue (a) and zinc(II) ions after reaction with xylenol orange (b) are presented.

**Figure 2:**
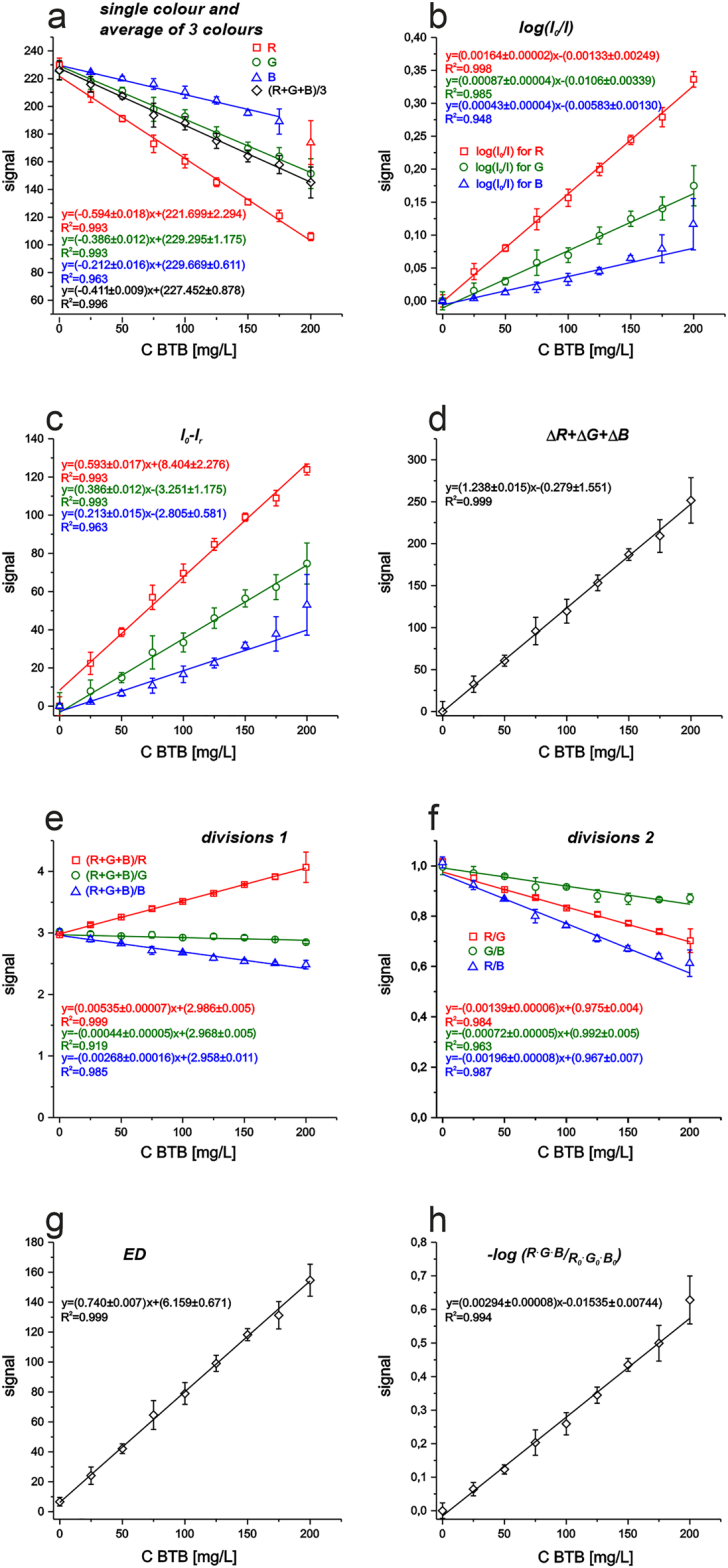
Calibration curves of µPADs for BTB detection obtained using different models of calculations: analytical signal taken as—(**a**) single colours and average of all 3 (R,G,B)—Eq. (1); (**b**) log(I_0_/I)—Eq. (2); (**c**) signal of blank subtract signal after reaction—Eq. (3); (**d**) sum of subtractions of each colour (R, G, B)—Eq. (4); (**e**, **f**) different division equations, e.g. Equation (5–5’’’) and others; (**g**) Euclidean distance—Eq. (6); (**h**) logarithm of multiplication of all colours for each standard divided by the same for blank—Eq. (7).

**Figure 3:**
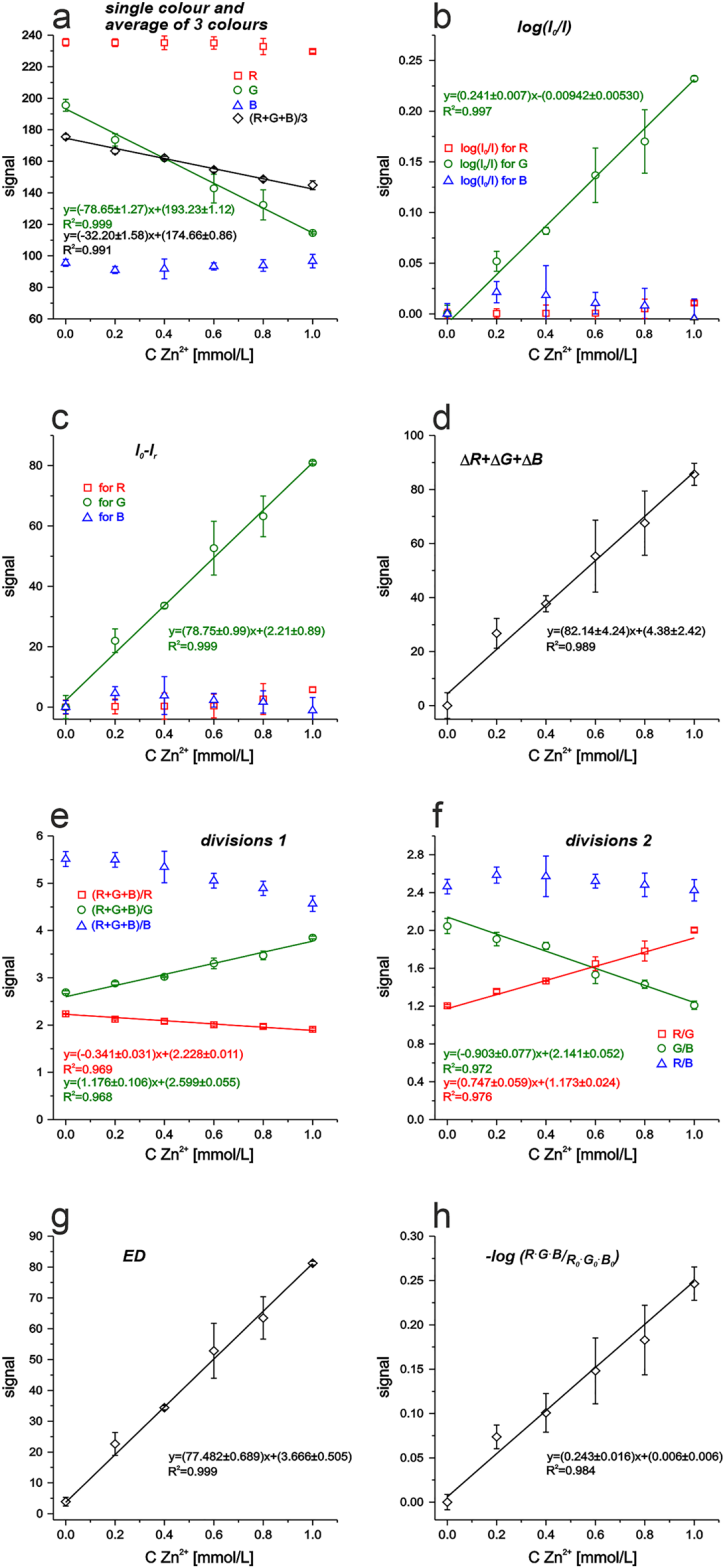
Calibration curves of µPADs for zinc(II) ions detection obtained using different models of calculations: analytical signal taken as—(**a**) single colours and average of all 3 (R,G,B)—Eq. (1); (**b**) log(I_0_/I)—Eq. (2); (**c**) signal of blank subtract signal after reaction—Eq. (3); (**d**) sum of subtractions of each colour (R, G, B)—Eq. (4); (**e**, **f**) different division equations, e.g. Equation (5–5’’’) and others; (**g**) Euclidean distance—Eq. (6); (**h**) logarithm of multiplication of all colours for each standard divided by the same for blank—Eq. (7).

Using the same image and values of R, G, and B (from the RGB colour space) obtained after images’ analysis using ImageJ software, several calibration graphs have been prepared (see Figs. 2, 3 for BTB and zin(II) ions, respectively). Different methods of mathematical data treatment have been compared.

As it can be seen from Fig. 2 depending on used mathematical approach, completely different sensitivities can be obtained. As the colour of model dye—bromothymol blue in pH = 9.2 is blue, the complementary colour on the colour wheel (Figure S3) is orange. Thus in RGB model, the red one is the closest colour, and it might be the first choice for BTB analysis (as it was mentioned before). On the other hand, the green one is also in similar distance to the complementary colour and the source colour. Moreover, the blue colour—the real one of the dye solution can also be considered. That is why without preparing all calibration graphs it is difficult to decide either single colour or any of other calculation methods is the optimal option.

On the other hand, in case of µPAD for zinc(II) determination, the obtained calibration curves presented in Fig. 3 clearly indicate that using single colour—red or blue is pointless. Other mathematical methods based on the green intensity only or with two other colours are worth deeper analysis. The colour of complex Zn^2+^-XO is orange-red to violet so also channel selection based only on the colour wheel might be a proper option (colour opposite to red is green). Nevertheless, to choose the best mathematical data treatment the comparison of all possibilities is necessary.

#### Analytical parameters

In standard analytical methods mathematical treatment of the raw data is important and can affect the final characterization of the method and accuracy of analyte determination. However, in microfluidic paper-based analytical devices, mathematical aspects are much more important. As it is seen from Table 3, the calculated sensitivities vary by even several orders of magnitude both, for bromothymol blue as well as zinc(II) ions detection. It is crucial to select the correct way of working up the results to be sure that small differences in concentration can be differentiated by the proposed method, and the determination of the analyte in unknown sample is accurate.

**Table 3:** The analytical parameters comparison between different methods of mathematical analysis of raw colour data.

BTB						Zn(II) ions					
No			Sensitivity (L mg^−1^)	Sensitivity error (%)	LOD (mg L^−1^)	No			Sensitivity (L mmol^−1^)	Sensitivity error (%)	LOD (mmol L^−1^)
1	Single colour	R	− 0.594 (± 0.018)	3.03	22.07	1	Single colour	R	–	–	–
2		G	− 0.386 (± 0.012)	3.11	16.54	2		G	− 78.65 (± 1.27)	1.61	0.12
3		B	− 0.212 (± 0.016)	7.55	40.13	3		B	–	–	–
4	$\frac{R + G + B}{3}$		− 0.411 (± 0.009)	2.19	13.62	4	$\frac{R + G + B}{3}$		− 32.20 (± 1.58)	4.91	0.15
5	$\log \left( {\frac{{I_{0} }}{I}} \right)$	R	0.00164 (± 0.00002)	1.22	11.84	5	$\log \left( {\frac{{I_{0} }}{I}} \right)$	R	–	–	–
6		G	0.00087 (± 0.00004)	4.60	24.66	6		G	0.241 (± 0.007)	2.90	0.12
7		B	0.00043 (± 0.00004)	9.30	66.06	7		B	–	–	–
8	$I_{0} - I_{r}$	R	0.593 (± 0.017)	2.87	22.07	8	$I_{0} - I_{r}$	R	–	–	–
9		G	0.386 (± 0.012)	3.11	16.54	9		G	78.75 (± 0.99)	1.26	0.12
10		B	0.213 (± 0.015)	7.04	55.14	10		B	–	–	–
11	ΔR + ΔG + ΔB		1.238 (± 0.015)	1.21	10.33	11	ΔR + ΔG + ΔB		82.14 (± 4.24)	5.16	0.16
12	$\frac{R + G + B}{R}$		0.00535 (± 0.00007)	1.31	6.79	12	$\frac{R + G + B}{R}$		− 0.341 (± 0.031)	9.09	0.25
13	$\frac{R + G + B}{G}$		− 0.00044 (± 0.00005)	11.36	92.59	13	$\frac{R + G + B}{G}$		1.172 (± 0.106)	9.01	0.21
14	$\frac{R + G + B}{B}$		− 0.00268 (± 0.00016)	5.97	46.79	14	$\frac{R + G + B}{B}$		–	–	–
15	R/G		− 0.00139 (± 0.00006)	4.32	27.59	15	R/G		0.747 (± 0.059)	7.90	0.13
16	G/B		− 0.00072 (± 0.00005)	6.94	73.65	16	G/B		− 0.903 (± 0.077)	8.53	0.22
17	R/B		− 0.00196 (± 0.00008)	4.08	40.45	17	R/B		–	–	–
18	ED		0.740 (± 0.007)	0.95	9.49	18	ED		77.482 (± 0.689)	0.89	0.09
19	$- \log \frac{{\left( {R \cdot G \cdot B} \right)}}{{\left( {R_{0} \cdot G_{0} \cdot B_{0} } \right)}}$		0.00294 (± 0.00008)	2.72	23.13	19	$- \log \frac{{\left( {R \cdot G \cdot B} \right)}}{{\left( {R_{0} \cdot G_{0} \cdot B_{0} } \right)}}$		0.243 (± 0.016)	6.58	0.17

Analysis of obtained calibration graphs for BTB (see Fig. 2) can give the information that different colour variants may be used for BTB determination. On the other hand, comparison of sensitivity values (see Table 3) seems to suggest that only two from nineteen studied methods of data analysis (no. 11 and 18—sum of subtractions and ED, respectively) should be considered for practical utility. Whereas, while adding into evaluation also the calculated sensitivity error and detection limit, also the third method ($\frac{R + G + B}{R}$, no. 12) should be taken into account. Moreover, this method offers the lowest detection limit. All three methods (no. 11, 12, 18) have similar sensitivity errors. Considered might be also the method no. 5 with similar sensitivity error, but with higher detection limit. Thus between these four mentioned computational methods the optimal one should be selected for a given experiment of BTB determination basing on the experimental needs (predicted analyte concentration in the sample as well as on the required resolution of differentiation the similar concentration values).

In case of zinc(II) ions complex with XO, from calibration curves of several options (see Fig. 3), only green channel taken into account for data treatment imply obtaining reasonable sensitivities and allow for potential practical usage. This conclusion is proved by analysis of the received sensitivities (Table 3), where G channel (in different mathematical approaches) or sum of differences, or ED (methods no. 2, 6, 9, 11, 18, respectively) might be analytically useful. In-depth analysis including sensitivity error and detection limit shows that the method no 11 offers high sensitivity, but its error is pretty high. Low detection limit and acceptable sensitivity error are offered by the methods no. 6 and 9. However, the lowest detection limit and the lowest sensitivity error is provided by ED method (no. 18), for which also the sensitivity was one of the highest.

Nevertheless, as comparing only sensitivities may be insufficient when selection of the optimal computational method, the delta signal ratio has been also calculated. This parameter contains the difference in signals for similar concentration values for each analyte divided by the average signals uncertainty. The comparison of these parameters is important because when comparing only the sensitivities, no attention is paid to the signal uncertainties, which may significantly affect the ability to distinguish similar concentration values. Final choice of the optimal method should be based on all aspects and adjusted to the given determination. Table 4 presents potential signals for two different, but similar concentration values of bromothymol blue and zinc(II) ions computed using proposed mathematical data processing methods. For some data it would be very difficult or even impossible to differentiate these analyte levels, whereas for some of them the results interpretation might be the piece of cake. It is worth mentioning that the high sensitivity, small sensitivity error and low detection limit are the most desirable in the Table 3. Whereas in the Table 4 the high delta signal ratio is preferable.

**Table 4:** Signal values for similar concentrations of bromothymol blue (60 and 65 mg/L) and zinc(II) ions (0.30 and 0.35 mmol/L) computed using proposed mathematical processing methods.

No	For BTB					For Zn^2+^ ions					
			60 mg/L	65 mg/L	ΔSignal ratio	No			0.30 mmol/L	0.35 mmol/L	ΔSignal ratio
1	Single colour	R	186.07 ± 3.41	183.10 ± 3.27	0.89	1	Single colour	R	–	–	–
2		G	206.16 ± 1.62	204.23 ± 1.56	1.21	2		G	169.63 ± 2.14	165.70 ± 1.99	1.90
3		B	216.87 ± 1.54	215.87 ± 1.69	0.62	3		B	–	–	–
4	$\frac{R + G + B}{3}$		202.77 ± 1.19	200.71 ± 1.16	1.75	4	$\frac{R + G + B}{3}$		165.00 ± 1.41	163.39 ± 1.31	1.18
5	$\log \left( {\frac{{I_{0} }}{I}} \right)$	R	0.097 ± 0.004	0.105 ± 0.004	2	5	$\log \left( {\frac{{I_{0} }}{I}} \right)$	R	–	–	–
6		G	0.041 ± 0.005	0.046 ± 0.005	1	6		G	0.063 ± 0.01	0.075 ± 0.01	1.20
7		B	0.020 ± 0.003	0.022 ± 0.004	0.57	7		B	–	–	–
8	$I_{0} - I_{r}$	R	43.96 ± 3.39	46.93 ± 3.25	0.89	8	$I_{0} - I_{r}$	R	–	–	–
9		G	19.89 ± 1.62	21.82 ± 1.57	1.21	9		G	25.84 ± 1.70	29.77 ± 1.57	2.40
10		B	10.00 ± 1.41	11.07 ± 1.55	0.72	10		B	–	–	–
11	ΔR + ΔG + ΔB		73.98 ± 2.19	80.17 ± 2.12	2.87	11	ΔR + ΔG + ΔB		29.02 ± 4.4	33.13 ± 4.19	0.96
12	$\frac{R + G + B}{R}$		3.307 ± 0.006	3.333 ± 0.006	4.33	12	$\frac{R + G + B}{R}$		2.13 ± 0.03	2.11 ± 0.02	0.80
13	$\frac{R + G + B}{G}$		2.942 ± 0.006	2.939 ± 0.006	0.50	13	$\frac{R + G + B}{G}$		2.95 ± 0.09	3.01 ± 0.09	0.67
14	$\frac{R + G + B}{B}$		2.797 ± 0.014	2.784 ± 0.013	0.96	14	$\frac{R + G + B}{B}$		–	–	–
15	R/G		0.892 ± 0.003	0.885 ± 0.003	2.33	15	R/G		1.40 ± 0.03	1.43 ± 0.02	1.20
16	G/B		0.948 ± 0.007	0.945 ± 0.006	0.46	16	G/B		1.87 ± 0.09	1.83 ± 0.09	0.44
17	R/B		0.849 ± 0.009	0.839 ± 0.009	1.11	17	R/B		–	–	–
18	ED		50.54 ± 1.09	54.24 ± 1.09	3.39	18	ED		26.91 ± 0.94	30.79 ± 0.88	4.26
19	$- \log \frac{{\left( {R \cdot G \cdot B} \right)}}{{\left( {R_{0} \cdot G_{0} \cdot B_{0} } \right)}}$		0.16 ± 0.01	0.18 ± 0.01	2	19	$- \log \frac{{\left( {R \cdot G \cdot B} \right)}}{{\left( {R_{0} \cdot G_{0} \cdot B_{0} } \right)}}$		0.079 ± 0.015	0.091 ± 0.015	0.80

± values are considered as the 95% lower and upper confidence limit of Y value. ΔSignal ratio as Δsignal divided by the average signals uncertainty.

Many of obtained signals (adding their uncertainties) are almost the same for both analysed values of concentration, e.g. for BTB the differences of signals for 60 mg/L and 65 mg/L calculated as logarithm of G and B intensities are, respectively, the same as or lower than the uncertainty of the signal (methods no. 6 and 7, respectively). Whereas, for some methods the signal ratios are large enough that these concentrations could most likely be distinguished using a given method. The example is the Euclidean distance for zinc(II) ions complex detection (method no. 18)—the Δsignal is more than four times higher than the calculated uncertainties of each signal. After analysis of data from Table 4 for bromothymol blue determination the sum of subtractions, Euclidean distance and $\frac{R + G + B}{R}$ might be considered for further experiments (methods no. 11, 12, and 18, respectively). Whereas for zinc(II) ions Euclidean distance (method no. 18) seems to be the best method for mathematical analysis and further determination. However, I_0 _− I_r_ of green channel intensities (method no. 9) might be also considered. The final chosen method should be in correlation with expected analyte concentrations in samples.

The comparison of data presented in Tables 3 and 4 shows that the choice of method made solely on the basis of sensitivity analysis (Table 3) may be insufficient to distinguish similar concentrations, and therefore insufficient for the analysis of real samples. Moreover, it is clear that it is impossible to clearly select one appropriate method for the interpretation and analysis of measurement data for all analytes before measurement and data processing. The results for both bromothymol blue and the complex of zinc(II) ions with xylenol orange show that a detailed mathematical analysis is necessary when working with µPADs and analyzing the colour. Even the order of magnitude of the obtained sensitivities when comparing the results for BTB and the zinc(II) ion complex is completely different.

Most of the methods used in literature for mathematical data treatment using RGB space are based on single colour intensity. Whereas in this study for BTB and zin(II) ions also more complex calculation methods were selected as ensuring better analytical parameters. Moreover, in the presented articles (Table 1) only some give an explanation of computational method choice is given, but mostly it is laconic, e.g. “based on experimental results” or “the highest intensities of X colour” without word which methods of data treatment were studied. Only in one article^23^ there was an information about among which methods (single colours of RGB and HSV colour space) the selection has been made. Nevertheless, wide comparison of the data treatment method was not made anywhere before.

Complex analysis of colour intensities of ROI is crucial in performing reliable mathematical data treatment in microfluidic paper-based analytical devices with colorimetric detection based on the RGB colour model. In many cases it is not enough to select one channel offering the highest sensitivity as it was suggested elsewhere^10^. In both analysed cases (see Table 4), for BTB and zinc(II) ions, using the Euclidean distance as an analytical signal seems to be the one of optimal options. It is in agreement with the information in Table 1—in many publications this mathematical data treatment method is popular. Nevertheless, it cannot be taken as standard for all colour reactions. It is crucial to check different methods and select the proper one for the exact measurement/analyte detection basing on the predicted analyte concentration in samples, necessity of differentiation of similar concentrations etc. For this reason the blank calculation sheet (Libre Office Calc) with ready-to-use commands, has been attached to this article (Supplementary Material 2). It is shared in an open access form to promote fast comparison of mathematical analysis methods of the obtained colour intensities in the RGB colour space. The extracted RGB values from e.g. ImageJ software has to be copied and other calculations will be done automatically (the parameters given by the ImageJ which should be copied are highlighted in the shared file). This approach (using the RGB dedicated computational sheet) will make optical detection used scanners (and e.g. smartphones) basing the RGB detection more efficient and accurate.

#### HSV colour space

As mentioned in the Introduction, alternatively to the RGB colour model, the HSV may be used. It needs calculations allowing for the transformation of the obtained R, G, and B intensities to H, S and V (equations needed to be used for these recalculations are given in^23^). Using shown equations is tedious and needs careful evaluation of R, G, and B channels to correctly choose one for H value calculation. Whereas for S and V intensities two step-computing is needed. Nevertheless, there is another faster way of receiving H, S, and V values—installing a dedicated plugin (Color Space Converter) for ImageJ software. This plugin allows performing transformations of a raw image into H, S, and V colour spaces from which signals can be separately read. Regardless of the chosen version, still receiving HSV intensities is more time and labour consuming.

Images of BTB and zinc(II) ions calibrations were converted using the abovementioned converter for ImageJ. The obtained calibration curves are presented in Figure S5, whereas the analytical parameters as well as resolution of similar concentrations differentiation are presented in Table S2 (Supplementary material). In case of BTB analysis using HSV colour space, the S value might be considered into evaluation while looking only at sensitivity and LOD. Though sensitivity error and mentioned LOD were higher than those chosen among computation methods based on RGB. However, delta signal ratio is much smaller than those of the methods 11, 12 and 18 from RGB colour space what exclude using HSV colour space for BTB determination. For zinc(II) ions the obtained LODs using H and S values are pretty good, but the sensitivity errors are higher than those of selected computational methods based on RGB model. While considering also the delta signal ratio the values obtained from H and S values are much lower than for methods 9 and 18 chosen as preferred ones for zinc(II) analysis.

## Conclusions

In this article different scanning methods and modes were examined, and the optimal one have been selected. The best results were obtained for the photo mode, with colour restoration, in tiff saving format. Resolutions of 300 and 600 dpi gave similar sensitivities so time that can be allocated for the exact measurement could be the crucial parameter in making a final decision (scanning with 300 dpi is faster than with 600 dpi, and the determination sensitivity is almost the same). Similarly with the saving format—if the file size is crucial, then better to choose jpeg saving format instead of tiff (even as tiff offers lightly higher sensitivities). Moreover, working up the obtained scans is equal to collecting intensities of Red, Green and Blue which are extracted from the images by the software.

The article focused also on comparison of a variety of mathematical attempts to manage data obtained from the scans. In many cases simple usage of single colour chosen based on the colour wheel and complementary colours rule might be insufficient. However, the selection of proper mathematical data handling method for the whole project is time- as well as labour-consuming. Nevertheless, this work is worth to be done. Tests and calculations performed within this article clearly indicate that there is no one method which is versatile for all purposes. For this reason, the open-source sheet for fast analysis of data using all presented mathematical analysis methods has been shared. Combination of careful selection of the mathematical data management (suggested in this article) with the proposed automatic image processing^20^ might be a useful tool for analyses done using microfluidic paper-based devices with colorimetric detection based on image processing. What is more, the investigations and conclusions described in this article would be useful also for other detectors (e.g. smartphones) implemented for working with µPADs.

### Supplementary Information

Supplementary Information 1.

Supplementary Information 2.

## Data Availability

All data generated or analysed during this study are included in this published article and if needed may be made available in other forms upon request. Moreover the raw data have been deposited at the RepOD repository, under deposition address: 10.18150/VBF1NC.
