# Supplementary material for: Mathematical processing of RGB data in microfluidic paper-based analytical devices
Source: Sci Rep. 2024 Jun 13;14:13635. doi: 10.1038/s41598-024-63546-2 (PMC11176306; doi:10.1038/s41598-024-63546-2)
Supplement: Supplementary file 1 — Supplementary Information 1. [file 41598_2024_63546_MOESM1_ESM.pdf]

# Mathematical processing of RGB data in microfluidic paper-based analytical devices

Marta Fiedoruk-Pogrebniak

University of Warsaw, Faculty of Chemistry, Pasteura 1, 02-093 Warsaw, Poland

(marta.fiedoruk@uw.edu.pl)

## Portable scanners

Nowadays, many models of portable scanners are characterized by the same or similar parameters in comparison to the desktop ones. In the Table S1 the office scanner used in this work (Epson L3151) was combined with some examples of portable scanners offered on the market.

*Table S1 - Comparison of the parameters offered by selected portable scanners available on the market with a desktop one used in the research.*

| Scanner - model | Scanning resolution [dpi] | Scanning speed (black) | Scanning speed (colour) | Saving formats | Wifi |
|---|---|---|---|---|---|
| Epson L3151 | 1200 x 2400 | 200 dpi; 11 sec | 200 dpi; 28 sec. | BMP, JPEG, TIFF, PDF | yes |
| | | | | | |
| Brother DS-740D | 1200 x 1200 | 300 dpi; 15 pages/min | 300 dpi; 15 pages/min | HPEG, PDF, PNG, TIFF, e-mail | no |
| Brother DS-820W | 1200 x 1200 | 300 dpi 7,5 pages/min | 300 dpi 7,5 pages/min | JPEG, TIFF, BMP, PNG, GIF, PDF, e-mail, cloud | yes |
| Brother DS-920DW | 1200 x 1200 | 7,5 pages/min | - | JPEG, TIFF, PDF, e-mail, cloud, SD | yes |
| IRIS IRIScan Book 5 | 1200 x 1200 | 1 sek | 2 sek | JPEG, PDF, MicroSD | no |
| FUJITSU ScanSnap iX100 | 600 x 600 | - | 5,2 sec, 300 dpi, 10 pages/min | JPEG, PDF, e-mail, cloud | yes |
| Epson WorkForce ES-60W | 600 x 600 dpi | 4 sec; 8,5 pages/min | 4 sec | PDF, JPEG, TIFF | yes |
| Canon image FORMULA P-208II | 600 x 600 | - | 16 pages/min | JPEG, TIFF, PDF, PNG, BMP, e-mail, cloud | (yes) – extra wifi module |

## Selection of the time gap for zinc(II) ions detection

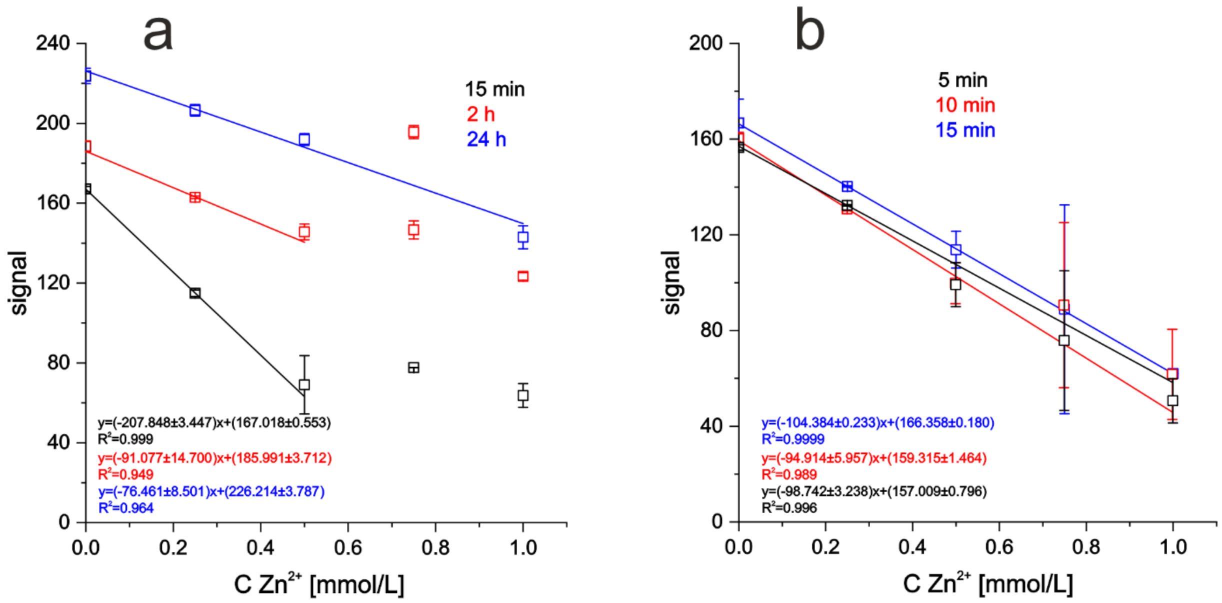


*Figure S1. Optimization of the time a) between reagent (xylenol orange) deposition and zinc(II) ion standard solutions, b) between zinc(II) ion standard solutions and detection*

## Scanning parameters

Scans with different scanning parameters ((i) resolution – 300/600 dpi, (ii) scanning mode – photo/document, (iii) exposure to radiation – low/medium/high, (iv) colour restoration – yes/no, (v) saving format – jpeg/tiff) were studied using BTB standard solutions. Obtained scans are presented in Figure S2 whereas received calibration curves are shown in Figure S4.

Analysis of scans obtained using different scan formats shown in Figure S1 leads to the conclusion that in some cases even naked eye observation can indicate those options that are clearer and provide more detail. Examples illustrating these differences include pairs of scans performed in photo and document scanning modes (comparing the same resolution and the same saving formats) as well as photo mode of scanning in both saving formats – jpeg and tiff, but with application of colour restoration while scanning. The comparison of scans (Fig. S2) with the developed numerical results and obtained sensitivities (Figure S4) allows to choose the most appropriate scanning method.

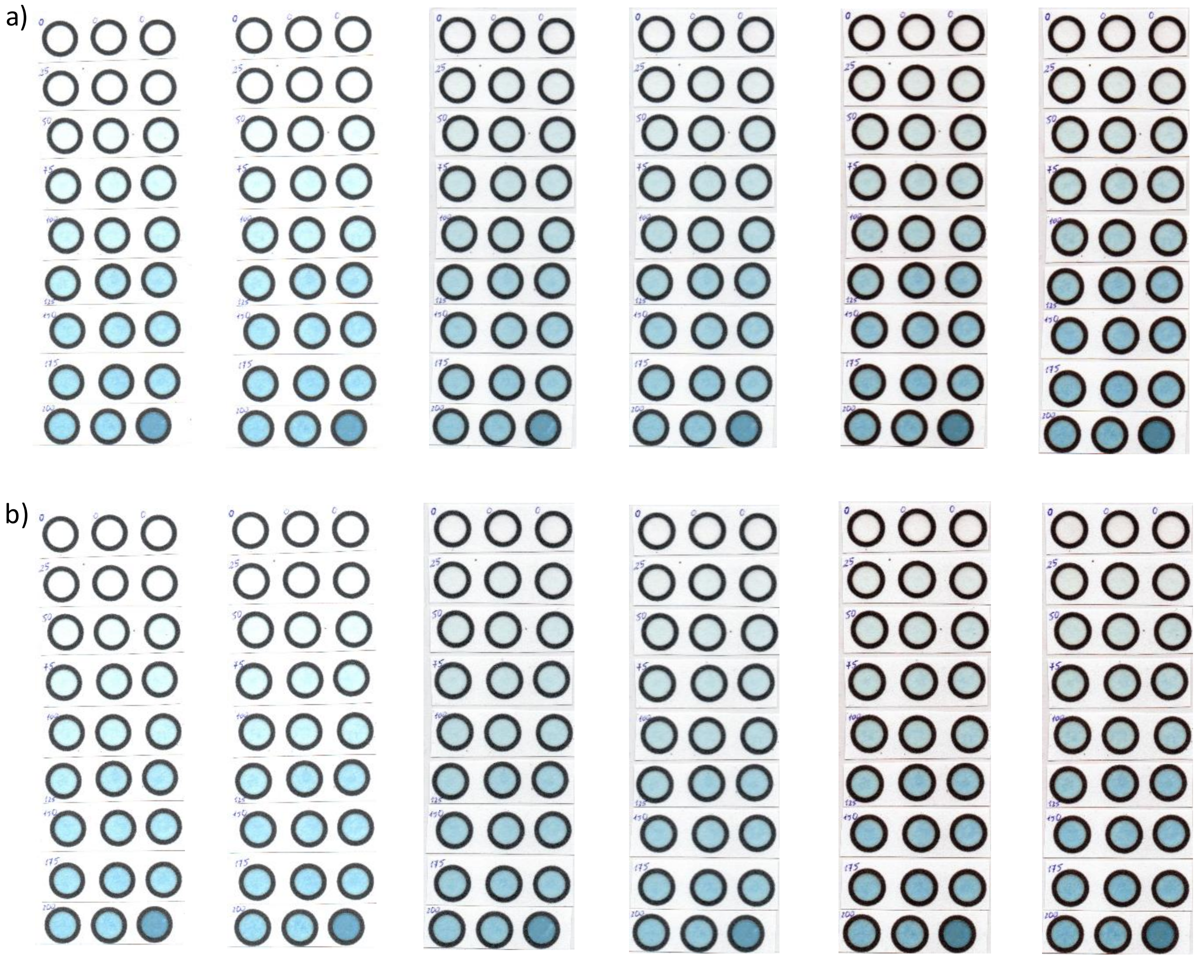

*Figure S2. Scans obtained with different scanning parameters: a) 300 dpi: order from the left side: document-jpeg, document-tiff, photo-jpeg without CR (colour restoration), photo-tiff- without CR, photo-jpeg with CR, photo-tiff-with CR, b) 600 dpi, order as in a).*

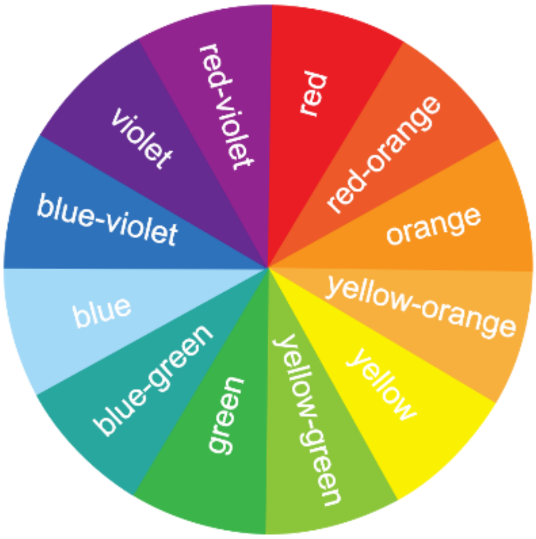


*Figure S3. The colour wheel.*

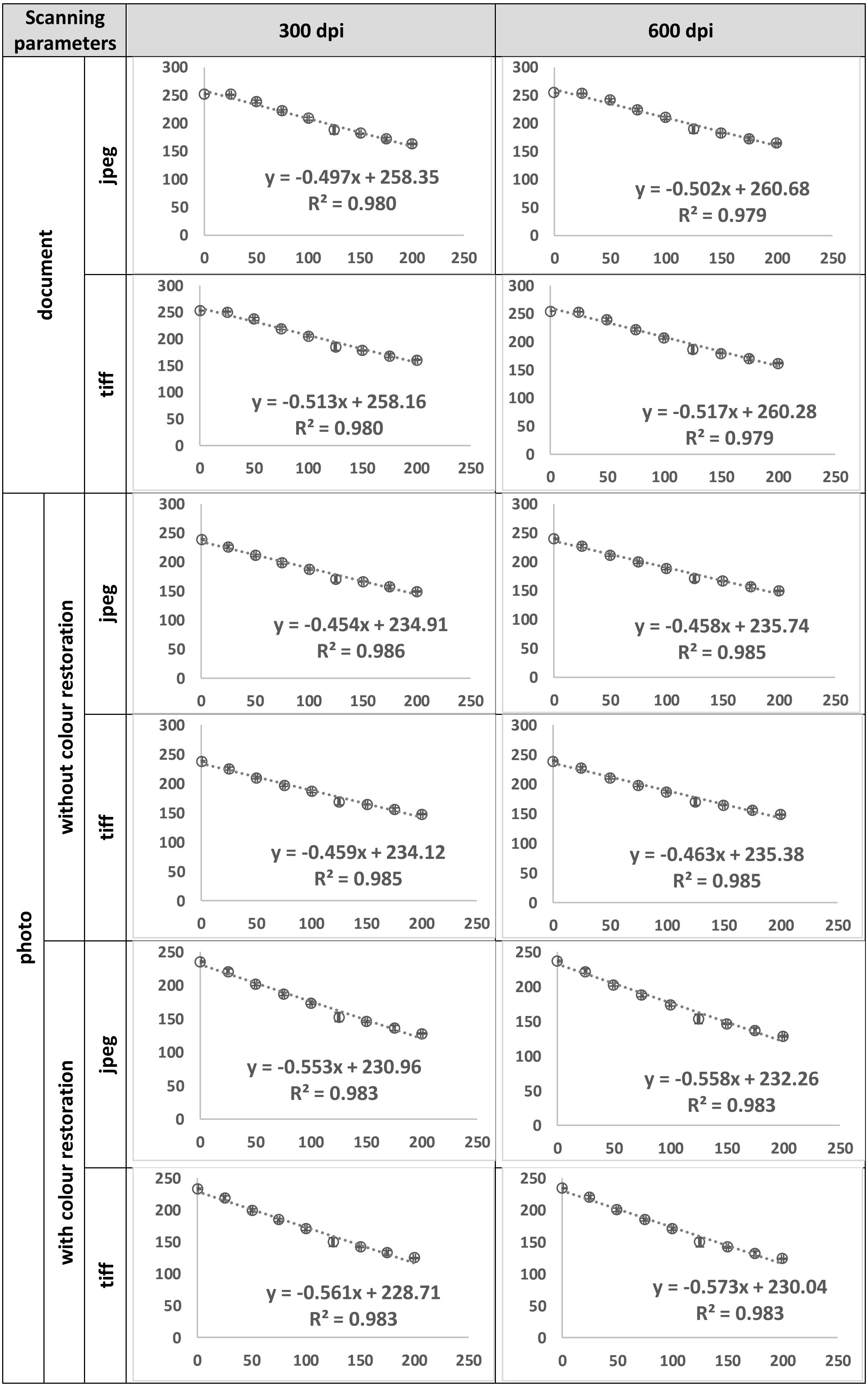


*Figure S4. Calibration curves for BTB obtained using different scanning parameters; OY axis – R intensity, OX axis – BTB concentration [mg/L].*

**HSV colour space**

The HSV intensities were collected from the converted images of BTB and zinc(II) ions calibrations using the Color Space Converter (https://imagej.net/ij/plugins/color-space-converter.html) – the plugin for the ImageJ. The obtained calibration curves are presented in Figure S5, whereas the analytical parameters as well as resolution of similar concentrations differentiation are presented in Table S2.

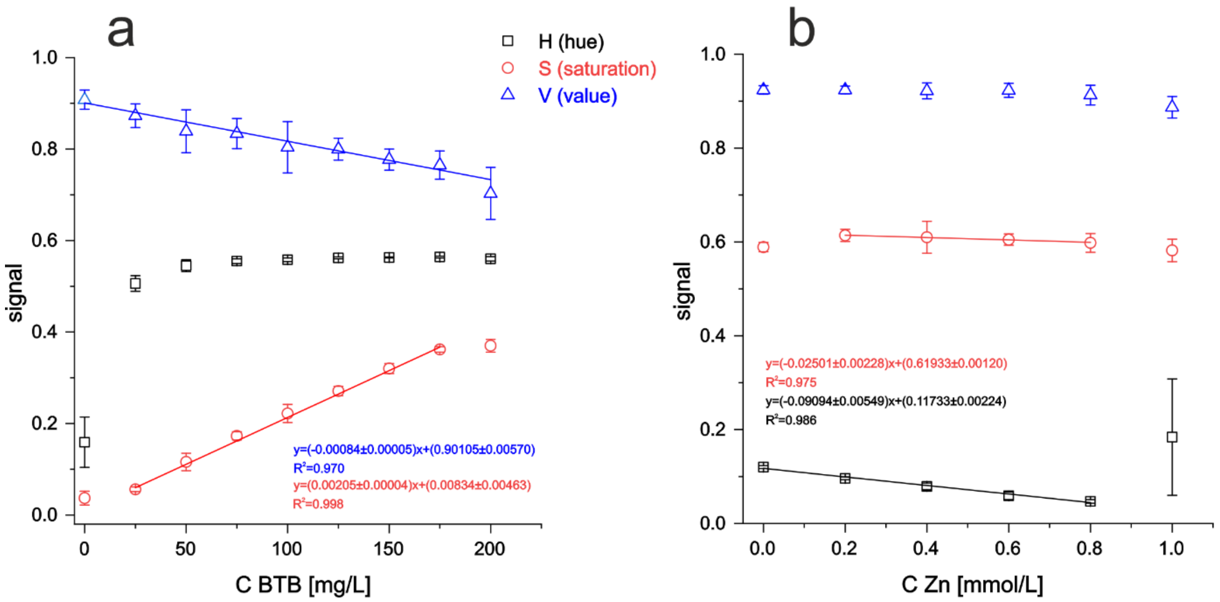


*Figure S5 - Calibrations curves for HSV colour space for a) BTB and b) zinc(II) ions.*

*Table S2 - The analytical parameters of HSV colour space analysis and signal values for similar concentrations of bromothymol blue (60 and 65 mg/L) and zinc(II) ions (0.30 and 0.35 mmol/L) computed using HSV model. ±values are considered as the 95% lower and upper confidence limit of Y value. ΔSignal ratio as Δsignal divided by the average signals uncertainty.*

| For BTB | | | | | For $Zn^{2+}$ ions | | | | |
|---|---|---|---|---|---|---|---|---|---|
| **No.** | | **Sensitivity [L·mg$^{-1}$]** | **Sensitivity error [%]** | **LOD [mg·L$^{-1}$]** | **No.** | | **Sensitivity [L·mmol$^{-1}$]** | **Sensitivity error [%]** | **LOD [mmol·L$^{-1}$]** |
| **1** | **H** | - | - | - | **1** | **H** | -0.09094 (±0.00549) | 6.04 | 0.13 |
| **2** | **S** | 0.00205 (±0.00004) | 1.95 | 10.57 | **2** | **S** | -0.02501 (±0.00228) | 9.12 | 0.13 |
| **3** | **V** | -0.00084(±0.00005) | 5.95 | 54.09 | **3** | **V** | - | - | - |

| **No.** | | **60 mg/L** | **65 mg/L** | **ΔSignal ratio** | **No.** | | **0.30 mmol/L** | **0.35 mmol/L** | **ΔSignal ratio** |
|---|---|---|---|---|---|---|---|---|---|
| **1** | **H** | - | - | - | **1** | **H** | 0.090 ± 0.005 | 0.086 ± 0.005 | 0.80 |
| **2** | **S** | 0.131 ± 0.007 | 0.141 ± 0.007 | 1.42 | **2** | **S** | 0.612 ± 0.003 | 0.611 ± 0.003 | 0.33 |
| **3** | **V** | 0.851 ± 0.009 | 0.847 ± 0.009 | 0.44 | **3** | **V** | - | - | - |
